# Mentors also need support: a study on their difficulties and resources in medical schools

**DOI:** 10.1590/S1516-31802012000400009

**Published:** 2012-09-04

**Authors:** Marina de Castro Nascimento Gonçalves, Patricia Lacerda Bellodi

**Affiliations:** I MSc. Psychologist, Postgraduate Program on Preventive Medicine, Faculdade de Medicina da Universidade de São Paulo (FMUSP), São Paulo, Brazil.; II PhD. Psychologist and Mentoring Program Coordinator, Faculdade de Medicina da Universidade de São Paulo (FMUSP), São Paulo, Brazil.

**Keywords:** Preceptorship, Mentors, Education, medical, Psychology, Qualitative research, Tutoria, Mentores, Educação médica, Psicologia, Pesquisa qualitativa

## Abstract

**CONTEXT AND OBJECTIVE:**

Mentors have been recognized as important elements in the personal and professional development of medical students. However, few investigations have sought to understand their development, needs and difficulties. Our objective was to investigate the perceptions of a group of mentors regarding difficulties experienced over time and the resources used to face up to them.

**DESIGN AND SETTING:**

Qualitative exploratory study on mentors at Faculdade de Medicina da Universidade de São Paulo (FMUSP). In the FMUSP Mentoring Program, mentors follow and guide students throughout the course, and are responsible for heterogeneous group of students, in relation to the academic year.

**METHOD:**

Semi-structured interviews were conducted with 14 FMUSP mentors.

**RESULTS:**

For many of the mentors, the difficulties related to initial doubts about the role, frustration with the students’ attendance and overloading of daily tasks. To address such difficulties, these mentors used external resources and their own life experience and personal way of dealing with situations. Some mentors did not perceive difficulties for themselves or for students.

**CONCLUSIONS:**

Like in other mentoring programs, many difficulties perceived by mentors seem to be derived from the context of medical education itself. However, unlike in other experiences, FMUSP mentors do not feel that there is lack of support for their role, since this is regularly provided in the structure and dynamics of the program. The “difficulty in perceiving difficulties”, presented by some mentors, demands further investigation for better and greater understanding.

## RESUMO

**CONTEXTO E OBJETIVOS:**

Mentores têm sido reconhecidos como elementos importantes para o desenvolvimento pessoal e profissional de estudantes de Medicina. Entretanto, poucas investigações buscam compreendê-los em seu desenvolvimento, necessidades e dificuldades. Nosso objetivo foi investigar as percepções de um grupo de mentores sobre as dificuldades vivenciadas ao longo do tempo e os recursos utilizados para enfrentá-las.

**TIPO DE ESTUDO E LOCAL:**

Estudo qualitativo e de caráter exploratório, com mentores da Faculdade de Medicina da Universidade de São Paulo (FMUSP). No Programa Tutores, mentores acompanham e orientam alunos ao longo do curso, sendo responsáveis por grupos heterogêneos de alunos em relação ao ano acadêmico.

**MÉTODO:**

Entrevistas semi-estruturadas foram realizadas com 14 mentores da FMUSP.

**RESULTADOS:**

Muitos mentores reconheceram como dificuldades as dúvidas iniciais com o papel, a frustração com a adesão dos alunos e a sobrecarga de tarefas do cotidiano. Para enfrentá-las, eles utilizam recursos externos, sua própria experiência de vida e o modo pessoal de abordar as situações. Uma parcela dos mentores não reconheceu dificuldades, para eles ou para os alunos.

**CONCLUSÕES:**

Como em outros programas, muitas das dificuldades percebidas pelos mentores mostram ser derivadas do próprio contexto da formação médica. Entretanto, diferentemente de outras experiências, os mentores FMUSP não se ressentem da falta de suporte para a sua função, estando este presente regularmente na estrutura e dinâmica do programa. A “dificuldade em perceber dificuldades”, apresentada por alguns mentores, demanda investigações posteriores para melhor e maior compreensão.

## INTRODUCTION

In Homer’s *Odyssey*, Mentor was a wise and faithful friend of Odysseus, King of Ithaca, to whom he entrusted the care of his son, Telemachus, while absent for the Trojan War. Mentor was responsible for the boy’s education and character development, guiding the decisions and choices to be made, and especially encouraging Telemachus to keep going at critical moments. In this task, Mentor was not alone: he received help as well. Pallas Athena, the goddess of wisdom and bright eyes, often assumed Odysseus’s friend’s form to “help him to help” the young Telemachus.^1^ Because of this relationship, the personal name Mentor has been adopted as a term meaning a trusted guide and counselor who supports and stimulates less experienced people in their personal and professional development.^2^

Today, mentors are recognized as an important element in the personal and professional development of young medical students. Mentoring programs have been introduced in medical schools,^3^ through recognition that the journey of future doctors is filled with demanding tasks, vicissitudes and challenges to overcome.

There is great concern in studies in this field to list and describe the attributes of a good mentor. Such individuals should be able to share their life experiences and technical expertise, have a respected professional standing and show genuine concern for others, with respect for their choices and interests. They should also be good communicators, ethical, flexible and available for long-term relationships.^4^^,^^5^^,^^6^^,^^7^^,^^8^ However, while a lot is expected of a mentor, a recent review of the literature^9^ showed that few investigations have been devoted to understanding mentors’ development, needs and difficulties.^10^^,^^11^

Considering that the mentoring relationship is inherently reciprocal, and that its satisfactory development depends on both mentor and mentee, investigations to understand mentoring from the mentors’ point of view are necessary for both practice and theoretical advances in this field.

At Faculdade de Medicina da Universidade de São Paulo (FMUSP), a Mentoring Program (Programa Tutores FMUSP) was implemented in 2001 for all its 1,080 students.^12^ The main goal of the Mentoring Program is to provide a mentor for medical students, who will follow them throughout the course, with regard to their personal and professional development. Each mentor is responsible for a heterogeneous group of 12 to 14 students, in relation to the academic year, with the aim of stimulating exchanges of experience between different phases of the course. Students’ participation is optional. There are some who always participate in the activities, others who sometimes participate and yet others who never do.

To be a mentor at FMUSP, it is necessary to be a physician actively involved in the educational context, to be close and empathetic to students’ needs and to have sufficient time and willingness to participate in initial training and supervision over time.

In order to understand the mentoring process from the mentor’s point of view, qualitative research was developed among a group of mentors at FMUSP, to explore their perceptions about their experiences over time.

## OBJECTIVES

This study aimed to present and, specifically, to discuss the difficulties and resources identified by mentors in performing their role and tasks.

## METHOD

Fourteen FMUSP mentors comprised the group of subjects investigated. In accordance with the methodological precepts of qualitative research,^13^ numerical representation was not sought in this study but, rather, quality of information in the interviews.

The study group was formed in such a way that it would be heterogeneous (intentional sampling) regarding demographic variables and experiences in the mentoring program. The following factors were taken into account: gender, age, specialty (clinical or surgical), length of time in the program (veteran: in the program since 2003; beginner: in the program since 2006), students’ attendance at mentoring meetings (satisfactory: out of the 10 planned meetings over the year, students attended five or more) and mentor’s attendance for supervision (satisfactory: out of the 10 supervision encounters of the year, mentors attended more than half of them).

It was deemed that the number of subjects included was adequate when it was perceived that redundancy of information or saturation of responses was starting to appear.

Through a semi-structured interview, the mentors responded to open-ended questions regarding their motivations, difficulties, support resources and changes over time.

In this study, the questions analyzed in order to understand mentors’ difficulties and resources were:

*- What are mentoring meetings with students like for you?*

*- When faced with any difficult situation in mentoring practice, you...*

*- What resources do you have for dealing with the difficulties encountered?*

Data were gathered during the second half of 2008 and in January 2009. During this period, the program comprised 81 mentors.

A total of 25 mentors were approached to participate in the study. Of these, four declined to be interviewed and five thanked us but said that they did not have time to schedule the interview meeting. A total of 14 interviews were conducted before data saturation was felt to have been reached.

All the interviews were conducted by the first author. The interviews lasted between 30 and 90 minutes and were conducted either in the teaching hospital or in the medical school, according to the interviewee’s preference.

The interviews were recorded and transcripts were analyzed using the technique of content analysis.^14^ Empirical categories were formulated after repeated reading of the data. The authors performed parallel readings of the material and met to discuss the construction and final definition of the categories, by consensus.

The study was approved by the FMUSP Ethics Committee, and the participants signed an informed consent statement.

## RESULTS

### Participants

Out of the 14 mentors interviewed, eight were male. The group age ranged from 35 to 61 years. Most of them were from a clinical medical specialty. Half of the mentors were veterans within the program, i.e. they had been doing this activity since its beginning. In the view of the majority of the mentors, the students’ attendance was unsatisfactory, i.e. out of the 10 planned meetings over the year, students had attended less than five. Similarly, most mentors poorly attended the supervisory meetings that were offered to them (Table 1).

**Table 1.** Characteristics of the mentors interviewed

Mentor	Gender	Age	Medical specialty	Length of time in the program	Students’ attendance to mentoring meetings	Mentors’ attendance at supervision meetings
1	M	55	Surgical	Veteran	Satisfactory	Satisfactory
2	F	52	Clinical	Beginner	Unsatisfactory	Unsatisfactory
3	F	49	Clinical	Veteran	Satisfactory	Unsatisfactory
4	M	48	Surgical	Veteran	Unsatisfactory	Unsatisfactory
5	M	61	Surgical	Veteran	Unsatisfactory	Unsatisfactory
6	M	35	Clinical	Beginner	Unsatisfactory	Satisfactory
7	F	54	Clinical	Veteran	Satisfactory	Unsatisfactory
8	M	44	Clinical	Beginner	Unsatisfactory	Unsatisfactory
9	M	40	Surgical	Beginner	Unsatisfactory	Unsatisfactory
10	F	50	Clinical	Veteran	Unsatisfactory	Unsatisfactory
11	F	46	Clinical	Beginner	Unsatisfactory	Satisfactory
12	F	50	Clinical	Veteran	Unsatisfactory	Unsatisfactory
13	M	37	Surgical	Beginner	Unsatisfactory	Unsatisfactory
14	M	50	Surgical	Beginner	Satisfactory	Satisfactory

## DIFFICULTIES

### I. Difficult situations? Yes!

#### Doubts at the beginning

Some mentors showed doubts about their task and role, which had been felt especially at the beginning of their experience:

*At first I was a little anxious because I had never done it before, and I was not sure what it would be like (Mentor 11);*

*I was a bit apprehensive, and felt anxious. I did not know exactly what it would be like or what would happen, so I scribbled several notes on a piece of paper about how I wanted the first time to be (Mentor 8);*

*I felt difficulty in knowing exactly what students wanted or how I had to show things to them, you know? (Mentor 2).*

#### Frustration with attendance

Many mentors highlighted frustration over time because of the students’ absence or their low attendance at the meetings:

*The first-year students come at the beginning and then they stop coming... We always expect we will see more people and if there are few of them, it is frustrating (Mentor 2);*

*It is useless to tell us that the number of students makes no difference. It is true that small groups work better, but it’s nice to see the group when all of the student come. I feel good about that (Mentor 8);*

*We are discouraged when they don’t come. “Is it my fault?” is the first question I asked myself. I thought they didn’t like me (Mentor 12).*

#### Daily tasks

An overloaded academic-professional agenda and limited time for the different activities were mentioned by several mentors as barriers to maintenance of a mentoring relationship:

*I’m always anxious because, beyond mentoring, I have to think about several other things during the day (Mentor 10);*

*I understand that it is hard for both mentors and students to be there during the mentoring period (Mentor 10);*

*The students always use the mentoring period to do something that, in their minds, is more important. The medical school overload and competition among students are very strong (Mentor 2).*

### II. Difficult situations? No…

#### What is a difficult situation?

Some mentors were unable to identify what exactly a difficult situation would be:

*Difficult? I don’t know if there was any difficult situation… Nothing has ever hit me directly. In every job, you have situations that are easy to solve and others that are more difficult. It is inherent to the job, right? (Mentor 7);*

*What is a difficult situation? I don’t know what you mean (Mentor 8).*

#### There are issues, but not problems

Other mentors considered the problems only from the students’ perspective. Even in that sense, they stressed that students did not bring serious problems to be solved, but issues to be discussed:

*I have not had difficult situations in mentoring. Students do not bring many problems to solve: they bring some questions, and sometimes they ask: what would you do in this situation? (Mentor 4);*

*Most students feel no need to seek help for serious problems. These are very rare. They come only with occasional doubts, mostly concerning medical school and careers (Mentor 5).*

## RESOURCES

### I. External resources

#### Program Coordination and Supervision

Program Coordination and Supervision was recognized by some mentors as an appropriate and welcoming environment for questions and guidance when difficulties were faced:

*I’m in contact with the program coordinator and I also take my doubts to the supervisor (Mentor 11);*

*I have a group of people who I know that I can rely on. In the mentoring program, there are many people who I can talk to about doubts and I’m absolutely secure because of this (Mentor 12).*

#### Exchanges with other mentors

Contact with other mentors at the time of supervision was also recognized as an important source of help, thus making it possible to share difficulties and experiences.

*We can talk to other colleagues and see if they have faced similar situations (Mentor 4);*

*The mentors send emails to one another, so I feel very comfortable in telling them everything (Mentor 10).*

### 2. Internal resources

#### My life experience

Many mentors identified their own personal or professional life experience as a resource for dealing with difficulties. They accessed past experiences that had been satisfactorily resolved, and adapted them to the present moment:

*I think that to be a mother helps in mentoring, I have three kids. In a way, the students are my children... (Mentor 3);*

*I think that to have worked under stressful situations, as I experienced in my medical practice, helps in mentoring... It’s like a school for life (Mentor 11);*

*The fact that I’m a pediatrician helps me a lot... (Mentor 12).*

#### My personal style

The personal way of dealing with situations also appeared as a resource in the mentors’ relationship with students and tasks.

*First, I have to understand the situation, and what is happening. Then I will try to solve it in the proper way (Mentor 7);*

*I always ask direct questions (Mentor 12);*

*I think the best resource I have is “feeling” (Mentor 13).*

## DISCUSSION

In this study, mentors were asked about their perceived difficulties and support resources in a mentoring program for an undergraduate medical course. Some mentors reported doubts and difficulty in dealing with the initial expectations about the role and the mentoring tasks. Such doubts, especially at the beginning of the experience, are understandable and natural. Mentors from other programs have also shown concerns about the role, and have presented anxiety and uncertainties about their personal skills. In the School of Medicine of the University of Washington,^11^ the mentors interviewed also reported that they were concerned about not doing enough for their students or not having personal skills for managing the meetings.

It is known that motivation, knowledge and experience as a doctor, teacher or researcher are not enough to be a good mentor or to establish a satisfactory mentoring relationship.^15^ In the FMUSP Mentoring Program, there is initial training, but the mentors interviewed showed that this is not enough, thus reinforcing the notion that in mentoring, “when you start working with your student, you’re never sure what will happen”.^16^

Over time, when mentoring meetings took place, the greatest difficulty perceived by most of the mentors was the students’ low attendance. Similar problems have been reported in other studies. In a program designed to support freshmen, mentors of the Ribeirão Preto Medical School (Faculdade de Medicina de Ribeirão Preto, FMRP), in Brazil, reported difficulty in motivating students to participate, and felt discouraged due to the students’ low attendance.^17^ Mentors of the Karolinska Institutet Teaching Hospital in Sweden have also reported that it is very easy for students to escape from contact and that the relationship with them did not become as deep as expected.^10^

Students’ attendance is a challenge for formal mentoring programs since it results from of a complex relationship that involves mentor, student and institution. It is known that mentors and students should have interpersonal skills and should understand and agree with the proposal. It is also necessary for the school to establish clear reasons why mentoring should take place and be prepared for it.^18^^,^^19^ However, there are many events and behavioral patterns in mentoring that cannot be predicted and controlled. Studies have recognized that the mentoring relationship is not like a formula: it develops naturally, and the most important element is the “chemistry” between mentor and student.^8^^,^^20^ The FMUSP Mentoring Program is characterized by students’ voluntary participation, which results in naturally heterogeneous attendance. Although the mentors understood the nature of students’ attendance in a rational manner, when the students did not attend the meetings or attended them in small numbers, this gave rise to feelings of frustration, anger and devaluation.

Another difficulty perceived by several mentors was their everyday academic and professional tasks and the pressures of time to accomplish all of them.

The many teaching, research and care activities are perceived by mentors as elements that create difficulties in mentoring relationships. This context, which is typical of medical teachers, leads them “to squeeze” mentoring into an agenda that is already full of commitments.^15^ The mentors also highlighted the students’ involvement in many activities due to the strong competition among them. In fact, studies on the FMUSP medical student profile have shown that 17% of freshmen already perceive the school to be very competitive. In the second year, this percentage rises to 51% and then increases to 65% in the final years.^21^ These mentors’ perceptions reinforce studies that indicate that the structure and dynamics of medical schools are barriers to mentoring, thereby negatively affecting the establishment of intense connections and long-term relationships between students and professors.^22^^,^^23^ While it is known that mentoring relationships go through phases^24^ and require commitment from both parties over time,^23^^,^^25^ and that regular interactions are significantly associated with satisfaction,^26^^,^^27^ the results from the present study show that there is a need for institutional strategies to protect the time allocated to mentoring meetings in medical schools.

There were mentors who did not identify any difficulty in being a mentor. For some of them, the situations experienced were regarded as inherent to the work; for others, there were no difficult situations to report because the students did not have problems. Stenfors-Hayes et al.^10^ also reported that mentors commonly presented the reaction that the job was easy because the students did not present problems, and thus concluded that solving problems was part of teachers’ expectations in mentoring.

The mentors’ perception of “no difficulty” in mentoring, for them or for students, can be understood in different ways. One understanding could include some characteristics traditionally associated with doctors and the medical culture, such as omnipotence and reluctance to seek help.^28^^,^^29^^,^^30^ Another could be that mentoring has a developmental goal and it is not necessarily focused on problems. Even though the academic life cycle is a process with some natural crises, this does not imply that these moments are synonyms for disasters. It can be also considered that physicians, because of their medical training, only consider a situation to be a “problem” when it is viewed as something involving significant risks, in the sense of “life or death”.

In relation to support resources, the mentors interviewed took a positive view of the coordination and supervision support offered by the FMUSP Mentoring Program. Lack of training is a common problem in mentoring programs and is one of the most frequently cited problematic outcomes for mentors within the medical context.^2^^,^^31^^,^^32^ However, from experience with mentors in residency medical programs, Freeman^33^ emphasized that training is only an initial induction phase. Continuing support is the next step because, as meetings develop, mentors find areas or attitudes that they need to examine, know more about and understand better.

The respondents also appreciated exchanges of experience with other mentors. This exchange of experiences made it possible to share coping strategies and could also promote what was observed by Dobie et al.:^11^ the development of a true “learning community” where everyone teaches and learns from others.

Other elements such as life experiences and personal ways of dealing with things were perceived by the mentors interviewed as important sources of support. It is not surprising that personal or professional experiences were considered by the mentors in their relationships with the students. By definition, mentors are precisely the kind of individual who through their histories can guide younger people at the beginning of their journeys. However, while past experience is important for mentors, on the other hand, they cannot forget that the “route” and “destination” will be determined by the young.^1^ In mentoring relationships, it is sometimes easy for mentors to develop a paternalistic attitude, and it is important not only be aware of this, but to resist it as well.^34^

The personal way of dealing with situations shows that personality, like in any other human relationship, is an element also present in mentoring. There are mentors who are more thinking-oriented, while others emphasize action and there are also those who are more focused on emotion and intuition. There are no studies on medical education, as far as we know, in which the influence of personality characteristics on mentoring relationships is examined. However, there is the recognition that a good mentoring relationship involves mentor self-awareness and identification of his own style, strengths and limitations. For a mentoring relationship to be effective, as highlighted by Taherian and Shekarchian,^34^ there is no requirement to incorporate any ideal attribute, but mentors need to have an open-minded attitude, with commitment and willingness to actively maintain the relationship over time.

## CONCLUSIONS

In this exploratory study, many mentors of an undergraduate medical program recognized difficulties in their activity: there were doubts at the beginning of the work, frustration with the students’ attendance over time and difficulty in inserting mentoring meetings into an overloaded work agenda. To address these difficulties, mentors used the support resources offered by the program, exchanged experiences with other mentors and also used their own life experience and personal way of doing things.

Other mentors in different mentoring programs within Medicine have reported similar difficulties, thus indicating that the context of medical training could be, through its very nature, a barrier to mentoring. However, unlike other programs in which mentors have demanded training and ongoing support, the FMUSP mentors perceived themselves to be supported by the program support resources, even if they did not participate very assiduously in the supervisory meetings.

This study also revealed, interestingly, that some mentors did not perceive difficulties in being a mentor. Such “difficulty in perceiving difficulties” is a result that requires further investigation in order to better understand the impact of this perception on mentoring relationships from the students’ perspective, as well as with regard to mentors’ own personal development.
